# Fragment-based design for the development of N-domain-selective angiotensin-1-converting enzyme inhibitors

**DOI:** 10.1042/CS20130403

**Published:** 2013-10-22

**Authors:** Ross G. Douglas, Rajni K. Sharma, Geoffrey Masuyer, Lizelle Lubbe, Ismael Zamora, K. Ravi Acharya, Kelly Chibale, Edward D. Sturrock

**Affiliations:** *Institute of Infectious Disease and Molecular Medicine, and Division of Medical Biochemistry, University of Cape Town, Observatory, Cape Town 7935, South Africa; †Department of Chemistry and Institute of Infectious Disease and Molecular Medicine, University of Cape Town, Rondebosch, Cape Town 7701, South Africa; ‡Department of Biology and Biochemistry, University of Bath, Claverton Down, Bath BA2 7AY, U.K.; §Lead Molecular Design, Sant Cugat del Vallès and Pompeu Fabra University, Barcelona, Spain

**Keywords:** angiotensin-1-converting enzyme (ACE), crystal structure, *in silico* screening, inhibitor design, kinetics, RXP407, ACE, angiotensin-1-converting enzyme, Ac-SDKP, *N*-acetyl-Ser–Asp–Lys–Pro, N-selective, N-domain selective, SHOP, scaffold hopping

## Abstract

ACE (angiotensin-1-converting enzyme) is a zinc metallopeptidase that plays a prominent role in blood pressure regulation and electrolyte homeostasis. ACE consists of two homologous domains that despite similarities of sequence and topology display differences in substrate processing and inhibitor binding. The design of inhibitors that selectively inhibit the N-domain (N-selective) could be useful in treating conditions of tissue injury and fibrosis due to build-up of N-domain-specific substrate Ac-SDKP (*N*-acetyl-Ser–Asp–Lys–Pro). Using a receptor-based SHOP (scaffold hopping) approach with N-selective inhibitor RXP407, a shortlist of scaffolds that consisted of modified RXP407 backbones with novel chemotypes was generated. These scaffolds were selected on the basis of enhanced predicted interaction energies with N-domain residues that differed from their C-domain counterparts. One scaffold was synthesized and inhibitory binding tested using a fluorogenic ACE assay. A molecule incorporating a tetrazole moiety in the P_2_ position (compound **33RE**) displayed potent inhibition (*K*_i_=11.21±0.74 nM) and was 927-fold more selective for the N-domain than the C-domain. A crystal structure of compound **33RE** in complex with the N-domain revealed its mode of binding through aromatic stacking with His^388^ and a direct hydrogen bond with the hydroxy group of the N-domain specific Tyr^369^. This work further elucidates the molecular basis for N-domainselective inhibition and assists in the design of novel N-selective ACE inhibitors that could be employed in treatment of fibrosis disorders.

## Abstract 2

The present paper reports the development of an N-domain selective ACE inhibitor and the molecular basis for the interaction of key active site residues with the ligand.

## INTRODUCTION

ACE (angiotensin-1-converting enzyme; EC 3.4.15.1) is a zinc dipeptidyl carboxypeptidase that plays a critical role in blood pressure regulation and electrolyte homoeostasis [1,2]. ACE consists of two catalytic domains (designated N- and C-domains) that, despite high-sequence identity and structural topology, display differences in substrate processing and inhibitor binding [3–6]. Studies involving the generation of mice containing one domain catalytically inactivated have provided important insight into the differing roles of the two domains. *In vivo* both domains clear the vasodilator peptide bradykinin with approximately equal efficiencies [7–9], the C-domain appears to be the prominent site for the production of vasoactive peptide angiotensin II, whereas the N-domain is the primary site for the clearance of tetrapeptide Ac-SDKP (*N*-acetyl-Ser–Asp–Lys–Pro) [8–10]. Ac-SDKP was first discovered owing to its ability to halt differentiation of the haematopoietic system [11,12]. More recent work has emphasized a potent anti-inflammatory and anti-fibrotic role in heart, liver, kidney and lung tissues [13–25]. Furthermore, Ac-SDKP levels have been shown to increase in patients acutely treated with ACE inhibitors [26] and suggests a possible therapeutic strategy for treating diseases involving fibrosis.

There are a number of ACE inhibitors that have been approved for clinical use [27]. These inhibitors inhibit both domains with similar affinity in the low nanomolar range [28]. While this allows for effective reduction in blood pressure, adverse drug effects result, possibly due to excessive bradykinin accumulation by dual domain blockade [29–31]. Thus selective inhibition of one ACE domain could allow for effective treatment with reduced adverse event incidence. More specifically, N-selective (N-domain-selective) inhibition could allow for the treatment of tissue injury and fibrosis diseases without affecting blood pressure and with reduced side effect profiles (Table 1).

**Table 1:** A summary of relevant ACE substrates and inhibitors

Domain contribution	Substrate	Biological action of substrate	Biological action of product after ACE hydrolysis	Inhibitor(s) targeting the indicated domain
Both domains	Bradykinin	Vasodilation	Inactive	Captopril, lisinopril, enalaprilat, ramiprilat and others [27]
approximately equally	Angiotensin-(1–7)	Vasodilation	Inactive	
C-domain specific	Angiotensin I	Inactive	Vasoconstriction, hypertrophy and fibrosis	RXPA380 [50], lisinopril-Trp [51], and inhibitors kAW and kAF [52]
N-domain specific	*N*-Acetyl-SDKP	Anti-fibrosis	Inactive	RXP407 [32]

RXP407 is a phosphinic peptidomimetic ACE inhibitor that displays approximately three orders of magnitude N-selectivity [32]. Detailed structure–activity studies revealed the importance of the *N*-acetyl group, P_2_ aspartate residue and C-terminal amide as contributors to the observed N-selectivity [32]. Using this information, we previously designed and synthesized ketomethylene inhibitors that incorporated the above functionalities either alone or in combination and showed that the incorporation of the P_2_ aspartate residue and *N*-protecting group resulted in a 1000-fold shift towards N-selectivity [33].

Elucidation of the ACE domain structures has allowed for the understanding of active site architecture and the relative positioning of amino acids that differ between the two domains [5,6]. Furthermore, the determination of the RXP407 co-crystallized with the N-domain shows that, of all the unique amino acids present, only prominent contacts with the unique residues Tyr^369^ and Arg^381^ (by the P_2_ aspartate residue) were exploited by the inhibitor [34]. This observation is consistent with a mutagenic study [35]. Structural information such as that above provides detailed binding modes of ligands in their respective active sites and can be utilized to generate scaffolds with similar or improved binding affinities with novel functionalities.

It was the purpose of the present study to exploit unique N-domain residue interactions with novel chemotypes using a receptor-based SHOP (scaffold hopping) GRID-based molecular modelling approach [36,37]. Such an approach has enabled the identification of potentially useful fragments that are not limited to natural amino acid residues. Furthermore, this approach has allowed for the production of a novel ACE inhibitor that is a potent inhibitor and displays approximately1000-fold N-selectivity.

## MATERIALS AND METHODS

### Modelling methodology and synthesis

**Figure 1:**
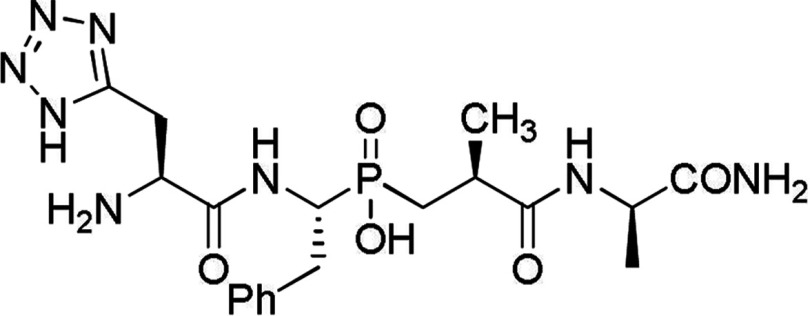
Structure of compound 33RE

SHOP methodology was employed to screen for commercially available building blocks that would possess similar or improved active site interaction properties (the procedure is outlined in the Supplementary Materials and methods section at http://www.clinsci.org/cs/126/cs1260305add.htm). Four amino acids differing in chemical nature from their C-domain counterparts were selected. Residues Tyr^369^ and Arg^381^ are located in the S_2_ subsite and have been shown by both mutagenesis and structural studies to be important for selective RXP407 binding [34,35]. These amino acids are replaced by Phe^391^ and Glu^403^, respectively, in the C-domain (all C-domain residues are given as tACE numbering). Thr^496^ is located on the border of the S_2_ and S_1_ subsites [6]. Owing to its proximity to the RXP407 Phe and the lack of hydrogen bonding potential of the corresponding C-domain residue (Val^518^), it was selected as a side-chain for further selective binding exploitation by hydrogen bonding. Thr^358^ is located in the S_2_′ subsite and differs from the corresponding C-domain residue Val^380^ [6]. As with Thr^496^, identification of functional groups that allows for specific interaction with the threonine side chain by hydrogen bonding could assist in generating N-selective inhibitors. Novel inhibitor structures that met the criteria were selected for further study. Final compound **33RE** (Figure 1) was synthesized using established synthetic approaches and purified to homogeneity (Supplementary Materials and methods section and Scheme S1 at http://www.clinsci.org/cs/126/cs1260305add.htm).

### Inhibition characterization

Parent molecule RXP407 and compound **33RE** were dissolved in sterile distilled water to yield stock solutions of 10 mM and subsequently serially diluted in assay buffer (50 mM Hepes, pH 6.8, 200 mM NaCl and 10 μM ZnSO_4_). Assays were performed on wild-type N-domain, C-domain and N-domain S_2_ mutants Y369F, R381E and YR/FE as described previously [32,35]. Briefly, 40 nM enzyme was incubated with an equivolume amount of an appropriate concentration range of phosphinic inhibitor at ambient temperature for 5 min. Enzyme-inhibitor solutions were then divided into aliquotes in triplicate 20 μl volumes followed by the addition of 280 μl of fluorogenic substrate (Abz)-FRK(Dnp)P-OH (in assay buffer) to yield a final substrate concentration of 4 or 8 μM. Residual enzyme activity was monitored continuously at *λ*_ex_=320 nm and *λ*_em_=420 nm using a fluorescence spectrophotometer (Cary Eclipse, Varian). Initial change of fluorescence over time was converted into arbitrary rate units and inhibitor-binding affinities determined using the Dixon method [38].

### Protein purification and X-ray crystallography

N-domain ACE was expressed and purified to homogeneity from CHO (Chinese-hamster ovary) cells [39]. The crystals of the N-domain ACE complex with 33RE were grown at 16°C by the hanging drop vapour diffusion method. N-domain ACE (5 mg/ml in 50 mM Hepes, pH 7.5) was pre-incubated with **33RE** (3.3 mM) at room temperature (20°C) for 2 h before crystallization. Pre-incubated sample (1 μl) was mixed with the reservoir solution consisting of 30% PEG550 MME/PEG20000, 100 mM Tris/Bicine, pH 8.5, and 0.06 M divalent cations (Molecular Dimensions), and suspended above the well. Crystals appeared within 3 days.

X-ray diffraction data for the N-domain ACE–**33RE** complex were collected on the PX station IO4-1 at Diamond Light Source (Didcot). A total of 720 images were collected using a Quantum-4 CCD (charge-coupled-device) detector (ADSC Systems). No cryoprotectant was used and the crystal was kept at constant temperature (100 K) under the liquid nitrogen jet during data collection. Raw data images were indexed and scaled with XDS [40] and the CCP4 program SCALA [41]. Initial phasing for structure solution was obtained using the molecular replacement routines of the program PHASER [42]. The atomic co-ordinates of N-domain ACE [34] (PDB code 3NXQ) were used as a search model. The resultant model was refined using REFMAC5 [43] and adjustment of the model was carried out using COOT [44]. Water molecules were added at positions where *F*_o_–*F*_c_ electron density peaks exceeded 3σ and potential H-bonds could be made. On the basis of the electron density interpretation, the inhibitor and glycosylated carbohydrate moieties were added in the complex structure and further refinement was carried out. The co-ordinate and parameter files for **33RE** were generated using SKETCHER [41] and validated through the PRODRG server [45]. Validation of the protein structure was conducted with the aid of MOLPROBITY [46]. Figures were drawn with PyMOL (DeLano Scientific). Hydrogen bonds were verified with the program HBPLUS [47]. The detailed refinement statistics for the complex structure are given in Table 2.

**Table 2:** Crystallographic statistics of N-ACE–33RE complex Values in parentheses are for last resolution shell.

Parameter	
Resolution (Å)	2.2
Space group	*P1*
Cell	a=73.1, b=77.3, c=82.9 α=88.4, β=64.3, γ=75.3
Number of molecules in ASU	2
Redundancy	3.9 (3.9)
Total/unique reflections	281360/73018
Completeness (%)	91.4 (88.7)
*R*_symm_*	12.5 (84.6)
*I*/σ(I)	10.5 (2.6)
*R*_cryst_†	18.8
*R*_free_‡	21.9
*R*_msd_ bond (Å)	0.008
*R*_msd_ angle (°)	1.18
B-factor analysis	
Protein all atom (chain A/B)	26.0/30.3
Protein main chain (chain A/B)	25.5/29.8
Protein side atoms (chain A/B)	26.4/30.8
Zinc ion (chain A/B)	17.6/14.2
Chloride ion (chain A/B)	17.4/22.7
Inhibitor (chain A/B)	21.3/18.2
Sugars (chain A/B)	47.6/59.1
Solvent	25.8
PDB code	4BXK

*R_symm_=Σ*_h_*Σ*_i_*[|*I_i_*(*h*)–<I(*h*)>|/Σ*_h_*Σ*_i_ I_i_*(*h*)], where *I_i_* is the *i*th measurement and <*I(h)>* is the weighted mean of all the measurements of *I*(*h*)

^†^*R*_cryst_=Σ*_h_|F_o_–F_c_|/*Σ*_h_F_o_,*where *F*_o_ and *F*_c_ are observed and calculated structure factor amplitudes of reflection *h* respectively

^‡^*R*_free_ is equal to *R*_cryst_ for a randomly selected 5% subset of reflections.

## RESULTS

**Figure 2:**
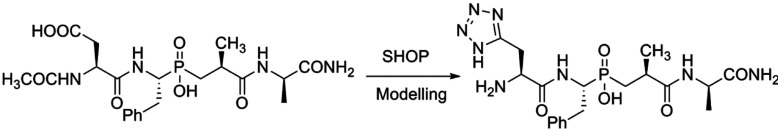
Fragment suggested to have complementary interactions with the protein in the same region as the one originally selected from 3NXQ [34]

**Figure 3:**
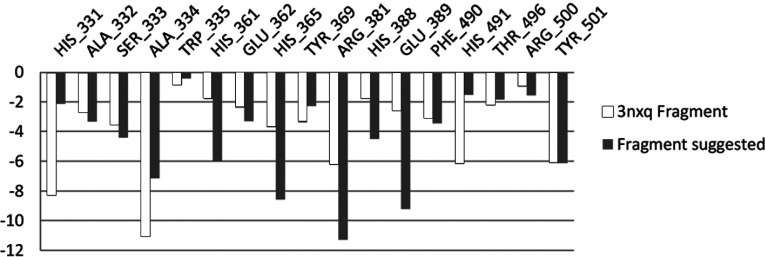
Interaction energies for all of the amino acids in the binding site for both RXP407[34] and compound 33RE

The SHOP modelling procedure yielded a list of fragments. Compounds containing novel functionalities were redocked into the N-domain active site. Those which had prominent interactions with identified amino acid side-chains were scored on the basis of interaction energies. This approach resulted in a short list of compounds that had interactions that were equal or improved compared to parent molecule RXP407 (Table 3). Compound **33RE** (Figure 1) was selected for synthesis and inhibition analysis given the prominence of the P_2_ position in conferring N-selectivity and the presence of a non-carboxylate moiety in this position (Figure 2). This fragment can replace the original one from RXP407 with similar interactions (Figure 3).

**Table 3:** Summary of results obtained from SHOP methodology *R*^2^ is given relative to the crystal structure RXP407 ligand backbone. Interaction energies of S_2_, S_1_ and S_2_′ amino acids of modified ligands compared with RXP407 are given in red, orange and purple respectively.

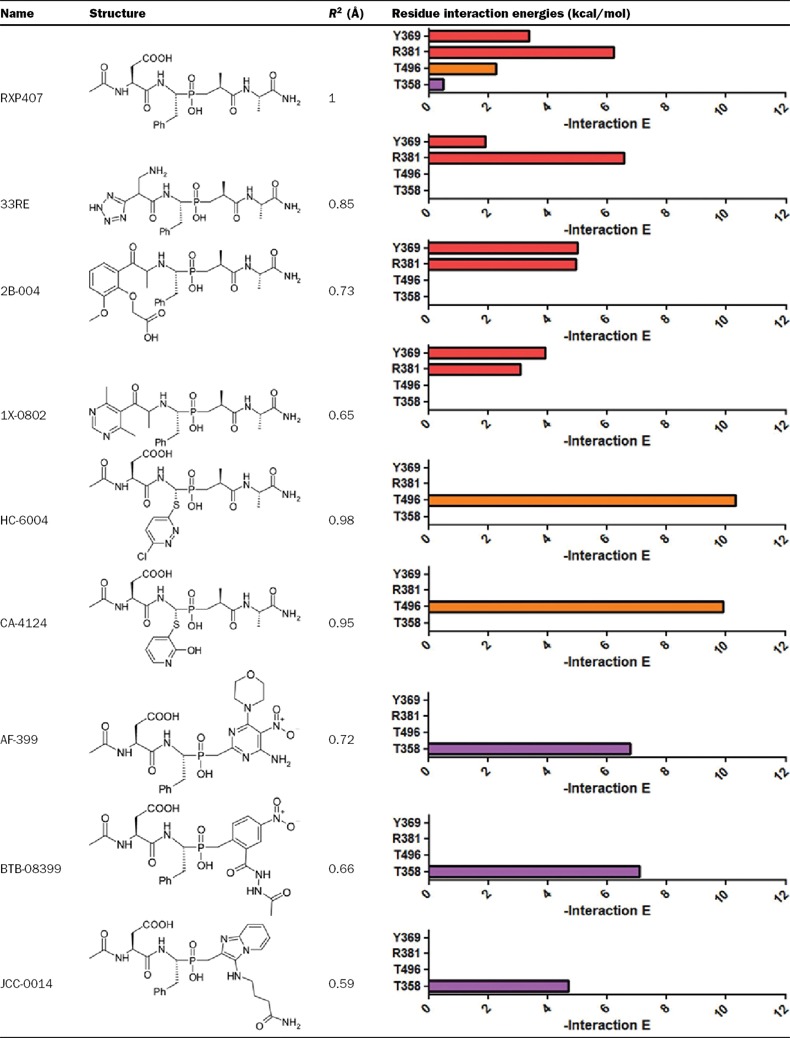

A classical and relatively straightforward 6-step synthetic approach resulted in the production of compound **33RE** in modest yields. Furthermore, this process possesses the possibility of scalability to larger amounts for further pharmacokinetic and preclinical testing.

**Figure 4:**
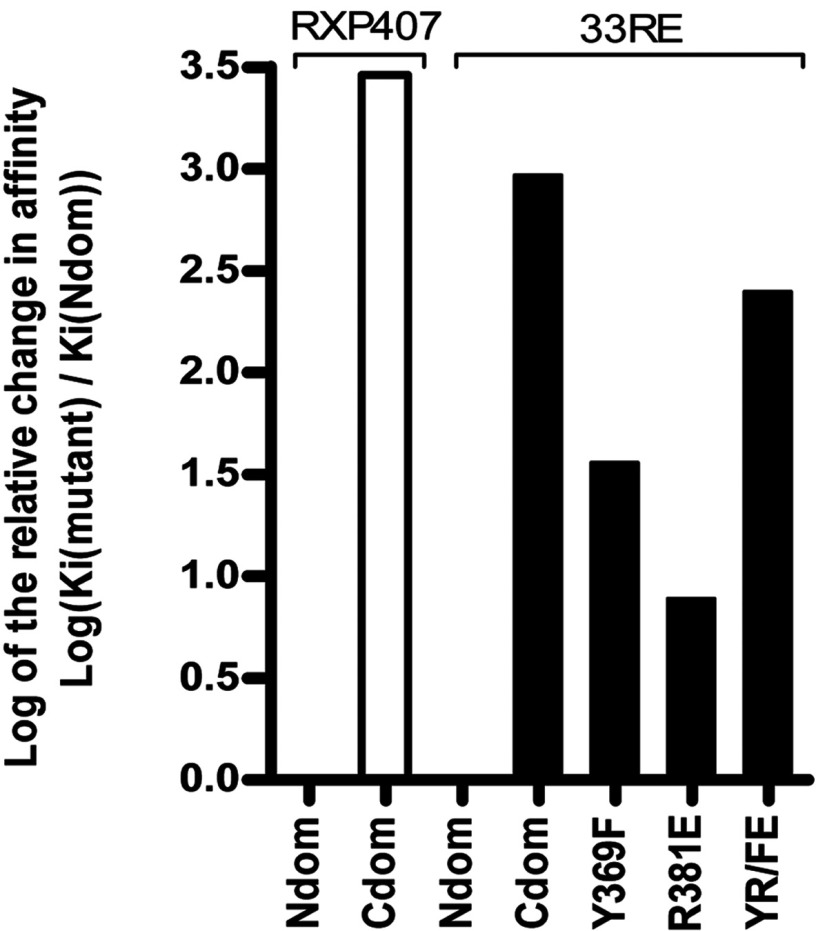
Logarithmic scale comparison of the relative 33RE-binding affinity of N-domain mutants with that of the wild-type domains (black bars) Relative binding affinity of wild-type domains for RXP407 is shown as white bars. Positive values represent decreased affinity relative to the unmutated N-domain, thus towards more C-domain-like *K*_i_.

Assessment of inhibitor potential was carried out using a continuous fluorogenic assay (Abz)-FRK(Dnp)P assay. Compound **33RE** displayed low nanomolar inhibition of the N-domain (*K_i_*=11.21±0.74 nM) in a similar range to parent molecule RXP407 (*K_i_=*21.03±0.27 nM). Characterization of the C-domain showed micromolar inhibition with compound **33RE** (*K_i_*=10 395±593 nM) as well as RXP407 (*K_i_*=60 826±9 175 nM), thus both compounds displayed three orders of magnitude N-selectivity (Table 4 and Figure 4). The active site mutants Y369F and R381E showed a marked decreased affinity for compound **33RE** (*K_i_*=404.4±17.25 nM and *K_i_*=86.97±6.29 nM, respectively) compared with wild-type N-domain. Upon substitution of both these residues in the double mutant YR/FE an additive effect was observed (*K_i_*=2794±156 nM), which led to a drastic decrease in **33RE** selectivity (only ~4-fold N-selectivity compared with 927-fold with wild-type). Given the importance of these two residues in conferring selectivity for both **33RE** and parent molecule RXP407, the kinetic results suggest that the actual interaction energies for the two inhibitors with these residues is critical [35].

**Table 4:** Inhibitor-binding constants (*K*_i_) determined for wild-type proteins and S_2_ mutants Values are means±S.E.M. C/N, C-terminal/N-terminal.

Construct	RXP407 *K*_i_ (nM)	Fold selectivity *K*_i_ (C/N)	33RE *K*_i_ (nM)	Fold selectivity *K*_i_ (C/N)
N-domain	21. 03±0.27	2896	11.21±0.74	927
C-domain	60 826±9 175		10 395±593	
Y369F	n.d.*	n.d.*	404.4±17.3	26
R381E	n.d.*	n.d.*	86.97±6.29	120
YR/FE	n.d.*	n.d.*	2794±156	4

*Not determined in the study [35].

**Figure 5:**
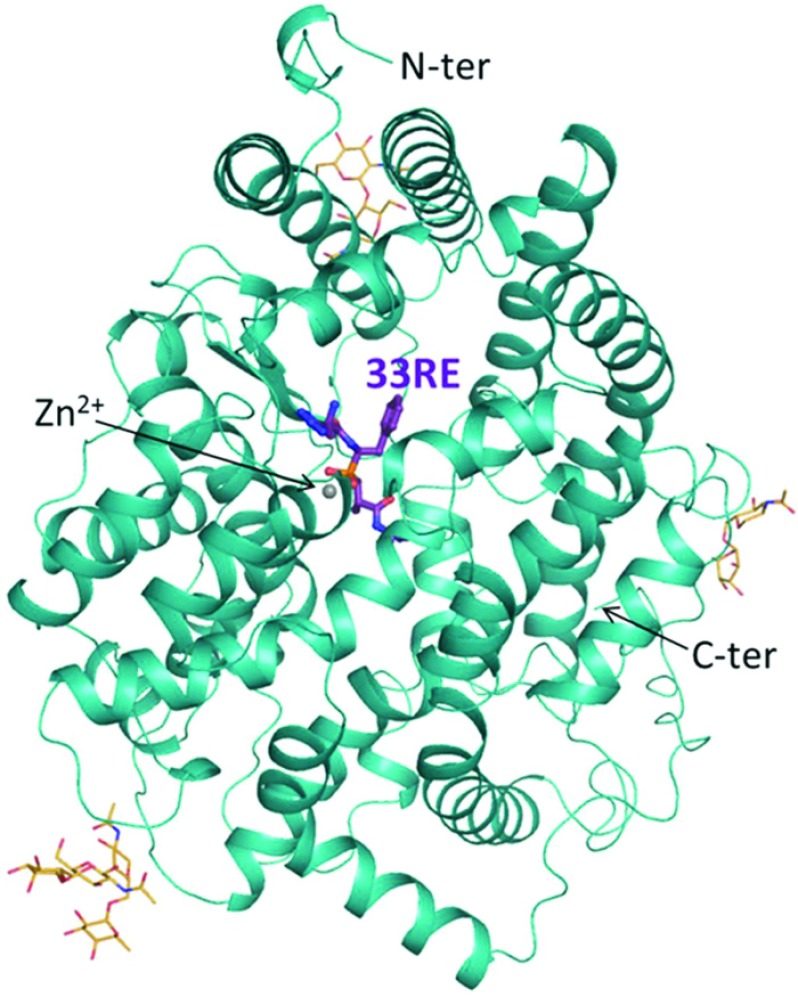
Crystal structure of N-domain ACE in complex with compound 33RE ACE is shown in cartoon representation (cyan) with catalytic zinc ion as a grey sphere, and glycosyl chains as orange sticks. Compound **RE33** is represented in purple.

**Figure 6:**
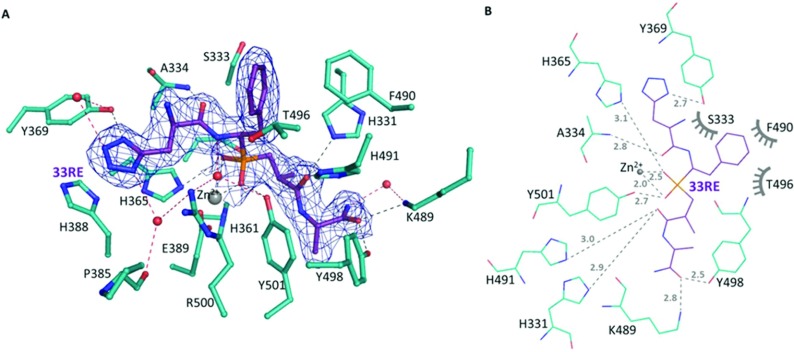
Binding of compound 33RE to N-domain ACE (**A**) Residues of N-domain ACE (cyan) interacting with **33RE** (purple) are shown as sticks. Potential hydrogen bonds and water-mediated interactions are shown as black and red dash lines respectively. Electron density (blue) corresponds to omit map for **33RE** at 1σ. Water and zinc ions are represented as red and grey spheres respectively. (**B**) Schematic diagram of potential hydrogen bonds (dashed lines, distances in Å: where 1 Å=0.1nm) and hydrophobic interactions (grey symbols) between N-domain ACE (cyan) and 33RE (purple) obtained from LIGPLOT [49].

The co-crystal structure of N-domain ACE was solved at 2.2 Å in complex with compound **33RE** (Figure 5). The crystals obtained were similar to those described by Anthony et al. [34] and diffracted in space group *P*1 with 2 molecules per asymmetric unit (Table 2). In both the molecules, clear electron density was visible for the entire ligand (Figure 6A), and allowed for a precise description of the molecular interactions responsible for inhibition. The backbone of **33RE** binds to N-domain ACE in a similar way to RXP407 with the central phosphate group co-ordinating with the catalytic zinc ion (Figure 6B and Table 5) and residues Tyr^501^ and His^365^. The mode of binding at the P_2_′ site is identical for the two inhibitors through hydrogen bonds with residues Lys^489^ and Tyr^498^, and also at the P_1_′ site where they can both make contact with His^331^ and His^491^. The P_1_ site is stabilized within the catalytic channel through hydrophobic interactions, particularly with Phe^490^, Ser^333^ and Thr^496^, and with a water-mediated interaction with Tyr^501^ and Arg^500^.

**Table 5:** Potential hydrogen bonds of 33RE binding to N-ACE

Residue	Atom	33RE	Distance (Å)
P2′			
Tyr^498^	OH	O	2.5
Lys^489^	NZ	O	2.8
Water 1	O	NAC	2.7
P1′			
His^331^	NE2	OAF	2.9
His^491^	NE2	OAF	3.0
Phosphate			
Tyr^501^	OH	OAH	2.7
	Zn^2+^	OAH	2.0
	Zn^2+^	OAI	2.5
His^365^	NE2	OAI	3.1
P1			
Water 2	O	NAV	3.0
P2			
Ala^334^	N	OAG	2.8
Tyr^369^	OH	NAS	2.7
Water 3	O	NAT	3.1
Water 4	O	NAR	3.1

**Figure 7:**
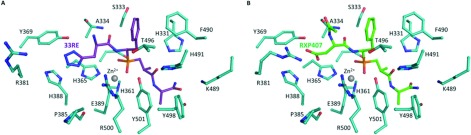
Comparison of the binding of compounds 33RE and RXP407 with N-domain ACE Residues of N-domain ACE (cyan) interacting with (**A**) **33RE** (purple) and (**B**) RXP407 (green; PDB code 3NXQ [34]) are shown as sticks.

The differences between compounds RXP407 and **33RE** reside at position P_2_. Although the backbone remains well anchored in S_2_ by a hydrogen bond with Ala^334^, modification of aspartate at P_2_ resulted in the loss of a potential water-mediated interaction with its C-terminal end and the amido group of Asp^336^. In addition, modification of the acidic side-chain caused the loss of the salt bridge with Arg^381^, which resulted in the reorientation of its long side chain away from the active site (Figure 7). However, inclusion of the tetrazole moiety allowed an alternative mode of binding of **33RE** through aromatic stacking with His^388^ and direct hydrogen bond with the hydroxy group of N-domain-specific Tyr^369^. Furthermore, a network of water molecules was clearly observed and stabilizes the compound in S_2_; particularly, water-mediated interactions were observed with Tyr^369^ on the one side and potential interactions with Glu^389^, Arg^500^ and Pro^385^ on the other side (Figure 6A).

## DISCUSSION

ACE inhibitors remain an effective therapeutic strategy in the treatment of cardiovascular disease. With the prominent anti-fibrotic and anti-inflammatory effects of Ac-SDKP evident, design of novel N-selective ACE inhibitors could be a possible approach in combating tissue fibrosis diseases without affecting blood pressure.

Structure-based drug design is one major approach in developing novel drug candidates. SHOP methodology, an approach whereby information regarding the ligand is utilized to identify novel chemotypes with similar chemical attributes, has been utilized previously to develop novel inhibitor scaffolds of thrombin, human immunodeficiency virus protease and influenza neuraminidase [36]. This approach has also been used to develop new 5-lipoxygenase inhibitors [48]. More recently, this method has been modified into the form of receptor-based SHOP to make use of the information of the protein–ligand complex [37]. In this approach, geometrical descriptions of the active site and co-crystallized ligand are used to substitute a position of the ligand with other known fragments. The feasibility of the approach was confirmed with cyclin-dependent kinase 2 scaffolds using an enrichment study and further novel scaffolds and inhibitors have been designed for p38 mitogen-activated protein kinase [37]. This approach was employed using N-selective ligand RXP407 to identify novel fragments that could be used in the design of N-selective ACE inhibitors.

Using the above receptor-based SHOP methodology, eight scaffolds were identified with known fragments that displayed equal or improved predicted interaction energies towards identified residues compared with RXP407. Specifically, the approach was able to identify fragments in the P_2_ position that had predicted interaction energies with Tyr^369^ and Arg^381^ similar to the RXP407 P_2_ aspartate residue. In the cases of Thr^496^ (S_1_ subsite) and Thr^358^ (S_2_′ subsite), where RXP407 has minimal contact with these unique N-domain residues, fragments were identified that had high predicted interaction energies. One such scaffold was synthesized using approaches that attempted to enhance the turnaround time of inhibitor production.

Assessment of inhibition was conducted using a fluorogenic (Abz)-FRK(Dnp)P assay. This analysis indicated that binding affinity of compound **33RE** to C- and N-domains, and therefore N-selectivity, is in the same range as that of the parent molecule. The data for RXP407 presented here compare well with previously published reports [32,35]. Since both compounds had similar predicted interaction energies for the residues of interest, it was anticipated that the N-selectivity of **33RE** would be comparable to RXP407. Kinetic analysis indicated three orders of magnitude N-selectivity for both compounds and serves as a proof of concept for using the discussed SHOP methodology in ACE inhibitor design. The co-crystal structure of N-domain ACE with **33RE** confirmed the interactions common to RXP407 and highlighted the importance of the P_2_ subsite for N-selectivity. The main difference in binding was seen in the loss of a salt bridge with Arg^381^ but enhanced interactions with Tyr^369^. Importantly, these two residues are not conserved in the C-domain (replaced by Glu^403^ and Phe^391^ respectively). Kinetic characterization revealed an 8-fold decrease in binding affinity for **33RE** upon mutating Arg^381^ to Glu, suggesting an alternative orientation of this residue than that seen in the crystal structure. A 36-fold decrease in binding affinity was observed for Y369F confirming the structural findings of a loss of direct and water-mediated hydrogen bonds to the P_2_ tetrazole of **33RE**. Thus, Tyr^369^ appears to be the major contributing residue towards the N-selectivity of compound **33RE**. The importance of the P_2_ subsite for selectivity was further emphasized by the additive effect observed in YR/FE constituting a 250-fold decrease in **33RE**-binding affinity compared with wild-type, similar to that seen previously for RXP407 [35]. Addition of the aromatic moiety also showed a stacking interaction with conserved residue His^388^, thus potentially improving affinity to both domains with a subsequent slight decrease in N-selectivity compared with parent molecule.

In conclusion, a modified SHOP methodology was employed to identify novel chemotypes with improved interactions with certain unique N-domain amino acid residues. A shortlist of scaffolds was identified and predicted to have equal or improved N-domain interactions compared with parent molecule RXP407. The most promising non-carboxylate P_2_ candidate (compound **33RE**) was synthesized and displayed potent and N-selective inhibition. This novel phosphinic ACE inhibitor provides a basis for incorporation of non-amino acid and non-carboxylate P_2_ functionalities into clinically relevant inhibitor backbones. It is noteworthy that a tetrazole moiety represents a bioisosteric replacement for a carboxylic acid group. N-domain selective inhibitors with drug-like characteristics could be useful in the treatment of tissue injury and fibrosis, which currently have limited treatment options.

## CLINICAL PERSPECTIVES

• The N-domain catalytic site of ACE is highly selective for the antifibrotic and anti-inflammatory tetrapeptide *N*-acetyl-Ser-Asp-Lys-Pro. Thus a fragment-based approach was used to design a novel inhibitor that was specific for the N-domain.
• A molecule incorporating a tetrazole moiety in the P_2_ position displayed potent inhibition of the N-domain and was 927-fold more selective for the N-domain than the C-domain. A crystal structure of the inhibitor in complex with the N-domain revealed its mode of binding through aromatic stacking with His^388^ and direct hydrogen bonding with the N-domain-specific Tyr^369^.
• This work further elucidates the molecular basis for N-domain-selective inhibition and assists in the design of novel N-selective ACE inhibitors that have potential for the treatment of fibrosis disorders

## Online data

Supplementary data

## References

[1] Acharya K. R., Sturrock E. D., Riordan J. F., Ehlers M. R. (2003). Ace revisited: a new target for structure-based drug design. Nat. Rev. Drug Discovery.

[2] Fyhrquist F., Saijonmaa O. (2008). Renin-angiotensin system revisited. J. Intern. Med..

[3] Soubrier F., Alhenc-Gelas F., Hubert C., Allegrini J., John M., Tregear G., Corvol P. (1988). Two putative active centers in human angiotensin I-converting enzyme revealed by molecular cloning. Proc. Natl. Acad. Sci. U.S.A..

[4] Wei L., Alhenc-Gelas F., Corvol P., Clauser E. (1991). The two homologous domains of human angiotensin I-converting enzyme are both catalytically active. J. Biol. Chem..

[5] Natesh R., Schwager S. L., Sturrock E. D., Acharya K. R. (2003). Crystal structure of the human angiotensin-converting enzyme–lisinopril complex. Nature.

[6] Corradi H. R., Schwager S. L., Nchinda A. T., Sturrock E. D., Acharya K. R. (2006). Crystal structure of the N domain of human somatic angiotensin I-converting enzyme provides a structural basis for domain-specific inhibitor design. J. Mol. Biol..

[7] Jaspard E., Wei L., Alhenc-Gelas F. (1993). Differences in the properties and enzymatic specificities of the two active sites of angiotensin I-converting enzyme (kininase II). Studies with bradykinin and other natural peptides. J. Biol. Chem..

[8] Fuchs S., Xiao H. D., Cole J. M., Adams J. W., Frenzel K., Michaud A., Zhao H., Keshelava G., Capecchi M. R., Corvol P., Bernstein K. E. (2004). Role of the N-terminal catalytic domain of angiotensin-converting enzyme investigated by targeted inactivation in mice. J. Biol. Chem..

[9] Fuchs S., Xiao H. D., Hubert C., Michaud A., Campbell D. J., Adams J. W., Capecchi M. R., Corvol P., Bernstein K. E. (2008). Angiotensin-converting enzyme C-terminal catalytic domain is the main site of angiotensin I cleavage *in vivo*. Hypertension.

[10] Rousseau A., Michaud A., Chauvet M. T., Lenfant M., Corvol P. (1995). The hemoregulatory peptide N-acetyl-SerAsp-Lys-Pro is a natural and specific substrate of the N-terminal active site of human angiotensin-converting enzyme. J. Biol. Chem..

[11] Lenfant M., Wdzieczak-Bakala J., Guittet E., Prome J. C., Sotty D., Frindel E. (1989). Inhibitor of hematopoietic pluripotent stem cell proliferation: purification and determination of its structure. Proc. Natl. Acad. Sci. U.S.A..

[12] Lombard M. N., Sotty D., Wdzieczak-Bakala J., Lenfant M. (1990). *In vivo* effect of the tetrapeptide, N-acetyl-Ser–Asp–Lys–Pro, on the G1–S transition of rat hepatocytes. Cell Tissue Kinet..

[13] Peng H., Carretero O. A., Brigstock D. R., Oja-Tebbe N., Rhaleb N. E. (2003). Ac-SDKP reverses cardiac fibrosis in rats with renovascular hypertension. Hypertension.

[14] Yang F., Yang X. P., Liu Y. H., Xu J., Cingolani O., Rhaleb N. E., Carretero O. A. (2004). Ac-SDKP reverses inflammation and fibrosis in rats with heart failure after myocardial infarction. Hypertension.

[15] Rasoul S., Carretero O. A., Peng H., Cavasin M. A., Zhuo J., Sanchez-Mendoza A., Brigstock D. R., Rhaleb N. E. (2004). Antifibrotic effect of Ac-SDKP and angiotensin-converting enzyme inhibition in hypertension. J. Hypertens..

[16] Peng H., Carretero O. A., Vuljaj N., Liao T. D., Motivala A., Peterson E. L., Rhaleb N. E. (2005). Angiotensin-converting enzyme inhibitors: a new mechanism of action. Circulation.

[17] Peng H., Carretero O. A., Liao T. D., Peterson E. L., Rhaleb N. E. (2007). Role of N-acetyl-seryl-aspartyl-lysyl-proline in the antifibrotic and anti-inflammatory effects of the angiotensinconverting enzyme inhibitor captopril in hypertension. Hypertension.

[18] Lin C. X., Rhaleb N. E., Yang X. P., Liao T. D., D’Ambrosio M. A., Carretero O. A. (2008). Prevention of aortic fibrosis by N-acetyl-seryl—aspartyl—lysyl–proline in angiotensin II-induced hypertension. Am. J. Physiol. Heart Circ. Physiol..

[19] Liao T. D., Yang X. P., D’Ambrosio M., Zhang Y., Rhaleb N. E., Carretero O. A. (2010). N-acetyl-seryl-aspartyl-lysyl-proline attenuates renal injury and dysfunction in hypertensive rats with reduced renal mass: council for high blood pressure research. Hypertension.

[20] Wang M., Liu R., Jia X., Mu S., Xie R. (2010). N-acetyl-serylaspartyl-lysyl-proline attenuates renal inflammation and tubulointerstitial fibrosis in rats. Int. J. Mol. Med..

[21] Rhaleb N. E., Pokharel S., Sharma U., Carretero O. A. (2011). Renal protective effects of N-acetyl-Ser-Asp-Lys-Pro in deoxycorticosterone acetate-salt hypertensive mice. J. Hypertens..

[22] Sun Y., Yang F., Yan J., Li Q., Wei Z., Feng H., Wang R., Zhang L., Zhang X. (2010). New anti-fibrotic mechanisms of n-acetyl-seryl–aspartyl–lysyl–proline in silicon dioxide-induced silicosis. Life Sci..

[23] Xu H., Yang F., Sun Y., Yuan Y., Cheng H., Wei Z., Li S., Cheng T., Brann D., Wang R. (2012). A new antifibrotic target of Ac-SDKP: inhibition of myofibroblast differentiation in rat lung with silicosis. PLoS ONE.

[24] Zhang L., Xu L. M., Chen Y. W., Ni Q. W., Zhou M., Qu C. Y., Zhang Y. (2012). Antifibrotic effect of N-acetyl-seryl-aspartyllysyl-proline on bile duct ligation induced liver fibrosis in rats. World J. Gastroenterol..

[25] Li P., Xiao H. D., Xu J., Ong F. S., Kwon M., Roman J., Gal A., Bernstein K. E., Fuchs S. (2010). Angiotensin-converting enzyme N-terminal inactivation alleviates bleomycin-induced lung injury. Am. J. Pathol..

[26] Azizi M., Rousseau A., Ezan E., Guyene T. T., Michelet S., Grognet J. M., Lenfant M., Corvol P., Menard J. (1996). Acute angiotensin-converting enzyme inhibition increases the plasma level of the natural stem cell regulator N-acetyl-seryl–aspartyl–lysyl–proline. J. Clin. Invest..

[27] Redelinghuys P., Nchinda A. T., Sturrock E. D. (2005). Development of domain-selective Angiotensin I-converting enzyme inhibitors. Ann. N.Y. Acad. Sci..

[28] Wei L., Clauser E., Alhenc-Gelas F., Corvol P. (1992). The two homologous domains of human angiotensin I-converting enzyme interact differently with competitive inhibitors. J. Biol. Chem..

[29] Nussberger J., Cugno M., Amstutz C., Cicardi M., Pellacani A., Agostoni A. (1998). Plasma bradykinin in angio-oedema. Lancet.

[30] Emanueli C., Grady E. F., Madeddu P., Figini M., Bunnett N. W., Parisi D., Regoli D., Geppetti P. (1998). Acute ACE inhibition causes plasma extravasation in mice that is mediated by bradykinin and substance P. Hypertension.

[31] Adam A., Cugno M., Molinaro G., Perez M., Lepage Y., Agostoni A. (2002). Aminopeptidase P in individuals with a history of angiooedema on ACE inhibitors. Lancet.

[32] Dive V., Cotton J., Yiotakis A., Michaud A., Vassiliou S., Jiracek J., Vazeux G., Chauvet M. T., Cuniasse P., Corvol P. (1999). RXP 407, a phosphinic peptide, is a potent inhibitor of angiotensin I converting enzyme able to differentiate between its two active sites. Proc. Natl. Acad. Sci. U.S.A..

[33] Sharma R. K., Douglas R. G., Louw S., Chibale K., Sturrock E. D. (2012). New ketomethylene inhibitor analogues: synthesis and assessment of structural determinants for N-domain selective inhibition of angiotensin-converting enzyme. Biol. Chem..

[34] Anthony C. S., Corradi H. R., Schwager S. L., Redelinghuys P., Georgiadis D., Dive V., Acharya K. R., Sturrock E. D. (2010). The N domain of human angiotensin-I-converting enzyme: the role of N-glycosylation and the crystal structure in complex with an N domain-specific phosphinic inhibitor, RXP407. J. Biol. Chem..

[35] Kroger W. L., Douglas R. G., O’Neill H. G., Dive V., Sturrock E. D. (2009). Investigating the domain specificity of phosphinic inhibitors RXPA380 and RXP407 in angiotensinconverting enzyme. Biochemistry.

[36] Bergmann R., Linusson A., Zamora I. (2007). SHOP: scaffold HOPping by GRID-based similarity searches. J. Med. Chem..

[37] Bergmann R., Liljefors T., Sorensen M. D., Zamora I. (2009). SHOP: receptor-based scaffold HOPping by GRIDbased similarity searches. J. Chem. Inf. Model.

[38] Dixon M. (1953). The determination of enzyme inhibitor constants. Biochem. J..

[39] Ehlers M. R., Chen Y. N., Riordan J. F. (1991). Purification and characterization of recombinant human testis angiotensin-converting enzyme expressed in Chinese hamster ovary cells. Protein Expr. Purif..

[40] Kabsch W. (2010). XDS. Acta Crystallogr., Sect. D: Biol. Crystallogr..

[41] Collaborative Computational Project Number 4 (1994). The CCP4 suite: programs for protein crystallography. Acta Crystallogr. Sect. D: Biol. Crystallogr..

[42] McCoy A. J., Grosse-Kunstleve R. W., Adams P. D., Winn M. D., Storoni L. C., Read R. J. (2007). Phaser crystallographic software. J. Appl. Crystallogr..

[43] Murshudov G. N., Vagin A. A., Dodson E. J. (1997). Refinement of macromolecular structures by the maximum-likelihood method. Acta Crystallogr., Sect. D: Biol. Crystallogr..

[44] Emsley P., Cowtan K. (2004). Coot: model-building tools for molecular graphics. Acta Crystallogr., Sect. D: Biol. Crystallogr..

[45] Schuttelkopf A. W., van Aalten D. M. (2004). PRODRG: a tool for high-throughput crystallography of protein-ligand complexes. Acta Crystallogr. D.

[46] Davis I. W., Leaver-Fay A., Chen V. B., Block J. N., Kapral G. J., Wang X., Murray L. W., Arendall III, W. B., Snoeyink J., Richardson J. S., Richardson D. C. (2007). MolProbity: all-atom contacts and structure validation for proteins and nucleic acids. Nucleic Acids Res..

[47] McDonald I. K., Thornton J. M. (1994). Satisfying hydrogen bonding potential in proteins. J. Mol. Biol..

[48] Aparoy P., Reddy K. K., Reddanna P. (2012). Structure and ligand based drug design strategies in the development of novel 5- LOX inhibitors. Curr. Med. Chem..

[49] WallacEe A. C., Laskowski R. A., Thornton J. M. (1996). LIGPLOT: a program to generate schematic diagrams of protein–ligand interactions. Protein Eng..

[50] Georgiadis D., Beau F., Czarny B., Cotton J., Yiotakis A., Dive V. (2003). Roles of the two active sites of somatic angiotensin-converting enzyme in the cleavage of angiotensin I and bradykinin: insights from selective inhibitors. Circ. Res..

[51] Nchinda A. T., Chibale K., Redelinghuys P., Sturrock E. D. (2006). Synthesis and molecular modeling of a lisinopriltryptophan analogue inhibitor of angiotensin I-converting enzyme. Bioorg. Med. Chem. Lett..

[52] Nchinda A. T., Chibale K., Redelinghuys P., Sturrock E. D. (2006). Synthesis of novel keto-ACE analogues as domainselective angiotensin I-converting enzyme inhibitors. Bioorg. Med. Chem. Lett..

