# Supplementary material for: Fragment-based design for the development of N-domain-selective angiotensin-1-converting enzyme inhibitors
Source: Clin Sci (Lond). 2013 Oct 22;126(Pt 4):305–13. doi: 10.1042/CS20130403 (PMC3875237; doi:10.1042/CS20130403)
Supplement: Supplementary data [file cs1260305add.pdf]

Clinical Science (2014) **126**, 305–313 (Printed in Great Britain) doi: 10.1042/CS20130403

Original Paper

SUPPLEMENTARY ONLINE DATA

Fragment-based design for the development of N-domain-selective angiotensin-1-converting enzyme inhibitors

Ross G. DOUGLAS*[1], Rajni K. SHARMA†[1], Geoffrey MASUYER‡, Lizelle LUBBE*, Ismael ZAMORA§, K. Ravi ACHARYA‡, Kelly CHIBALE† and Edward D. STURROCK*

*Institute of Infectious Disease and Molecular Medicine, and Division of Medical Biochemistry, University of Cape Town, Observatory, Cape Town 7935, South Africa
†Department of Chemistry and Institute of Infectious Disease and Molecular Medicine, University of Cape Town, Rondebosch, Cape Town 7701, South Africa
‡Department of Biology and Biochemistry, University of Bath, Claverton Down, Bath BA2 7AY, U.K.
§Lead Molecular Design, Sant Cugat del Vallès and Pompeu Fabra University, Barcelona, Spain

Clinical Science www.clinsci.org

MATERIALS AND METHODS

SHOP search

The methodology to perform a SHOP search consists of several steps [1]. (i) A database of fragments with the potential to replace P_2–P_2' groups was built. The database was prepared using commercially available building blocks: Aldrich, Bionet, CombiBlocks Enamine, Maybridge, Specs and Synthonix. The commercial structures were submitted to a virtual reaction algorithm, where each of the hypothetical interactions of the compounds is replaced by an anchor point, following the reaction pattern. The virtual compounds built in this way were converted into 3D and a conformational analysis performed using the algorithm implemented in SHOP. For each final structure a set of descriptors were computed: Shop-Geom, Shop-GRID, Shop-Shape and Shop-Finger. (ii) The database is screened using the ligand–receptor complex (PDB code 3NXQ; N-domain crystallized with inhibitor RXP407) [2]. The fragment shown in Figure S1 was the one used to define the region in the protein where the computation was carried out. (iii) A descriptor-based similarity analysis was performed comparing the query and the fragments in the database, and generating a list of ranked fragments that may replace the query. In this procedure, once the fragment has been selected in the ligand, it was removed from the ligand–receptor complex and a GRID computation in the protein cavity performed (Figure S2). There are four parameters that can be modified during this process: protein filtering (PF) option: the 'none' level was chosen in this computation which means that no atom is deleted from the protein; the grid clearance was set to 2 Å in all searches done; the cut-off parameter was set to 1.5 Å; and ligand filtering (LF) was set to one.

In Figure S2 the interaction fields in the regions of interest are shown. Four amino acids differing in chemical nature from their C-domain counterparts were selected. Residues Tyr^{369} and Arg^{381} are located in the S_2 subsite and have been shown by both mutagenesis and structural studies to be important for selective RXP407 binding [2,3]. These amino acids are replaced by Phe^{391} and Glu^{403}, respectively, in the C-domain (all C-domain residues are given as tACE numbering). Thr^{496} is located on the border of the S_2 and S_1 subsites [4]. Owing to its proximity to the RXP407 phenylalanine residue and the lack of hydrogen bonding potential of the corresponding C-domain residue (Val^{518}), it was selected as a side chain for further selective binding exploitation by hydrogen bonding. Thr^{358} is located in the S_2' subsite and differs from the corresponding C-domain residue Val^{380} [3]. As with Thr^{496}, identification of functional groups that allow for specific interaction with the Thr side chain by hydrogen bonding could assist in generating N-selective inhibitors. (iv) The selected fragments were then aligned with the query and placed in the protein cavity. If there were no collisions with the protein atoms (within a certain tolerance), the fragment was selected for further analysis. (v) Finally, the newly built molecule was optimized in the protein cavity and the calculation of the energy of interaction between the new ligand and the amino acids in the protein was performed by translating every atom from the ligand into a GRID atom type and calculating the interaction using the POSI directive in GRID.

At the end of the modelling process, the poses were confirmed visually. Top ranking molecules that had reasonable poses (compared with previous structures) were selected for further study.

Synthesis

(*R/S*)-2-methyl-3-(((*R*)-1-(*N*-(benzyloxycarbonyl)amino)-2-phenylethyl)-hydroxyphosphinyl)propanoic acid, ethylester **(1)** The commercially available (*R*)-((1-(*N*-benzyloxycarbonyl)

[1]**These authors contributed equally to this work.**

Correspondence: Professor Edward D. Sturrock (email edward.sturrock@uct.ac.za), Professor Kelly Chibale (email kelly.chibale@uct.ac.za) or Professor K. Ravi Acharya (email K.R.Acharya@bath.ac.uk).

© 2014 The Author(s)
The author(s) has paid for this article to be freely available under the terms of the Creative Commons Attribution Licence (CC-BY) (http://creativecommons.org/licenses/by/3.0/) which permits unrestricted use, distribution and reproduction in any medium, provided the original work is properly cited.

amino)-2-phenylethyl)phosphinic acid (571 mg, 1.78 mmol, 1 equiv.) and hexamethyldisilazane (2.1 ml, 8.9 mmol, 5 equiv.) were heated at 110 °C for 2 h under argon, then cooled to 60 °C. At this temperature ethyl methacrylate (0.335 ml, 2.67 mmol, 1.5 equi.) was added dropwise and the resulting solution was stirred for 3 h at 90 °C. The reaction mixture was cooled to 60 °C, methanol (5 ml) was added dropwise, and stirred at 25 °C for 20 min. The mixture was concentrated and the residue was dissolved in a mixture of 5% aqueous $NaHCO_3$ (10 ml) and diethyl ether (15 ml).The organic phase was separated and the aqueous phase washed twice with ether (10 ml × 2). The aqueous phase was acidified with 5% aqueous HCl to pH 1–2, the compound extracted with ethyl acetate (15 ml × 3), and combined organic phase dried with $MgSO_4$ and concentrated under vacuum to produce a white solid. Purification by silica gel chromatography using dichloromethane/methanol (8:2) as eluent, afforded diasteroisomeric mixture (4:2) of (*R/S*)-2-methyl-3-(((*R*)-1-(*N*-(benzyloxycarbonyl)amino)-2-phenylethyl)-hydroxyphosphinyl)propanoic acid, ethylester **1** (520 mg) as white solid with 67% yield.^{1}H NMR(300 MHz, $CDCl_3$): δ 7.30-7.16(10H, m, Ar-H), 5.33(1H, d, 9.1 Hz, -NH), 4.97(2H, s, -O$C\underline{H_2}$Ph), 4.27–4.02(3H, m, C$\underline{H}$PCH$_2$, O$C\underline{H_2}$CH$_3$), 3.30-3.24(1H, m, PCHCH$\underline{H}$Ph), 2.92-2.80(2H, m, PCHC$\underline{H}$HPh, PCH$_2$C$\underline{H}$), 2.28-2.19(1H, m, PCH$\underline{H}$CH), 1.83–1.74(1H, m, –PC$\underline{H}$HCH), 1.25 (3H, d, *J* 6.9 Hz, CHPCH$_2$CH(C$\underline{H_3}$)), 1.21(3H, d, *J* 7 Hz, OCH$_2$C$\underline{H_3}$); ^{13}C NMR(100 MHz, $CDCl_3$): δ 175.5, 156.1, 136.7, 136.3, 129.1(2C), 128.5(2C), 128.4, 128.2(2C), 127.8(2C), 126.7, 66.9, 61, 52.2, 33.8, 30.8, 29.9, 18.9, 14.1; ^{31}P NMR (162 MHz, $CDCl_3$): δ 52.3, 50.5; LC-ESI–MS(+ve): *m/z* 434.2$[M+H]^+$.

(*R/S*)-2-methyl-3-(((*R*)-1-(*N*-(benzyloxycarbonyl)amino)-2-phenylethyl)-adamantyloxyphosphinyl)propanoic acid, ethylester **(2)**.

To a refluxing solution of phosphinate **1** (600 mg, 1.38 mmol, 1 equiv.) and 1-adamantyl bromide (357 mg, 1.65 mmol, 1.2 equiv.) in chloroform (20 ml) silver (I) oxide (383 mg, 1.65 mmol, 1.2 equiv.) was added portionwise over 50 min. This solution was refluxed for an additional 2 h and then concentrated. The residue was treated with diethyl ether (20 ml), filtered through celite and concentrated. The residue was purified by silica gel chromatography using dichloromethane/isopropanol (95:5) as eluent and yielded a mixture of (*R/S*)-2-methyl-3-(((*R*)-1-(*N*-(benzyloxycarbonyl)amino)-2-phenylethyl)-adamantyloxyphosphinyl)propanoic acid, ethylester **2** (657 mg, 84%) as a colourless liquid.

^{1}H NMR(300 MHz, $CDCl_3$): δ 7.29–7.20(10H, m, Ar-H), 4.96(2H, s, –O$C\underline{H_2}$Ph), 4.17–4.09(3H, m, C$\underline{H}$PCH$_2$, O$C\underline{H_2}$CH$_3$), 3.29-3.23(1H, m, PCHCH$\underline{H}$Ph), 2.89–2.80(2H, m, PCHC$\underline{H}$HPh, PCH$_2$C$\underline{H}$), 2.38-2.29(1H, m, PCH$\underline{H}$CH), 2.17-2.06(9H, m, C$\underline{H}$ of Ad group, C$C\underline{H_2}$ of Ad group), 1.74-1.62(7H, m, PC$\underline{H}$H, C$C\underline{H_2}$CH of Ad group), 1.25 (3H, d 6.9 Hz, CHPCH$_2$CH(C$\underline{H_3}$)), 1.21(3H, d, *J* 6.9 Hz, OCH$_2$C$\underline{H_3}$); ^{13}C NMR (100 MHz, $CDCl_3$): δ 166.9, 155.9, 137, 136.4, 129.3, 129.2(2C), 128.4, 128(2C), 127.9(2C), 127.8, 126.6, 81.7, 66.9, 60.8, 52.7, 44.5(3C), 35.7(3C), 34.1, 31.2(3C), 30.8, 30.7, 19.2, 14.1; ^{31}P NMR (162 MHz, $CDCl_3$): δ 47.5, 46.3; LC-ESI–MS(+ve): *m/z* 568.2$[M+H]^+$.

(*R/S*)-2-methyl-3-(((*R*)-1-(*N*-(benzyloxycarbonyl)amino)-2-phenylethyl)-adamantyloxyphosphinyl)propanoic acid **(3)**.

The solution of pseudo dipeptide **2** (100 mg, 0.176 mmol) in methanol (5 ml) was treated with 4 M aqueous NaOH solution (0.4 ml) and the reaction mixture was stirred for 2 h at room temperature. The solvent was removed and the residue was diluted with H_2O (10 ml) and acidified with 5% aqueous HCl in an ice-water bath to pH 2. The aqueous phase was extracted with EtOAc ethyl acetate (20 ml × 2) and the combined organic layer was dried with $MgSO_4$ and concentrated to yield a mixture of (*R/S*)-2-methyl-3-(((*R*)-1-(*N*-(benzyloxycarbonyl)amino)-2-phenylethyl)-adamantyloxyphosphinyl)propanoic acid **3** (70 mg, 74%) as a white solid.

^{1}H NMR(300 MHz, $CDCl_3$): δ 7.30–7.14(10H, m, Ar-H), 4.90(2H, s, –O$C\underline{H_2}$Ph), 4.24–4.20(1H, m, C$\underline{H}$PCH$_2$), 3.18-3.13(1H, m, PCHCH$\underline{H}$Ph), 2.83–2.76(2H, m, PCHC$\underline{H}$HPh, PCH$_2$C$\underline{H}$), 2.37–2.25(1H, m, PCH$\underline{H}$CH), 2.11–1.97 (9H, m, C$\underline{H}$ of Ad group, C$C\underline{H_2}$ of Ad group), 1.74–1.62(7H, m, PC$\underline{H}$H, C$C\underline{H_2}$CH of Ad group), 1.19(3H, d *J* 7.1Hz, CHPCH$_2$CH(C$\underline{H_3}$)); ^{13}C NMR(100 MHz, DMSO): δ 177, 156.3, 138.6, 137.6, 129.3, 128.4(4C), 128(2C), 127.6(2C), 126.6, 81.7, 65.6, 54.7, 44.3(3C), 35.8(3C), 33.8, 31(3C), 30.5, 29.6, 19.6; ^{31}P NMR(162 MHz, DMSO): 47.5, 47.5; LC-ESI–MS(+ve): *m/z* 540.2$[M+H]^+$.

[(*R/S*)-2-methyl-{3-(((*R*)-1-(*N*-(benzyloxycarbonyl)amino)-2-phenylethyl)-adamantyloxyphosphinyl)propanoyl}amino]-(*S*)-2-propanamide (**4**).

To a solution of pseudo phosphinic acid **3** (480 mg, 0.892 mmol, 1 equiv.) in dichloromethane (5 ml), EDC.HCl (188 mg, 0.981 mmol, 1.1 equiv.), L-alaninamide. HCl (122 mg, 0.981 mmol, 1.1 equiv.), HOBt (129 mg, 0.981 mmol, 1.1 equiv.), DIPEA (0.311 ml, 1.78 mmol, 2 equi.) were added at 0 °C, and the mixture was stirred for 18 h at room temperature. H_2O was added and the aqueous phase was extracted with ethyl acetate (30 ml×3). The combined organic layer was washed with H_2O (10 ml), brine (10 ml), dried over $MgSO_4$ and concentrated. The residue was purified over silica gel chromatography using 5% dichloromethane/methanol as eluent and afforded [(*R/S*)-2-methyl-{3-(((*R*)-1-(N-(benzyloxycarbonyl)amino)-2-phenylethyl)-adamantyloxyphosphinyl)propanoyl}amino]-(*S*)-2-propanamide **4** (0.543 g, 73%) as a yellow liquid.

^{1}H NMR(300 MHz, $CDCl_3$): δ 7.27–7.16 (12H, m, Ar-H, CON$\underline{H_2}$), 4.97(2H, s, –O$C\underline{H_2}$Ph), 4.49–4.30(1H, m, C$\underline{H}$CONH$_2$), 4.21–4.16(1H, m, C$\underline{H}$PCH$_2$), 3.22-3.11(1H, m, PCHCH$\underline{H}$Ph), 2.79–2.67(2H, m, PCHC$\underline{H}$HPh, PCH$_2$C$\underline{H}$), 2.31-2.22(1H, m, PCH$\underline{H}$CH), 2.14–2.07 (9H, m, C$\underline{H}$ of Ad group, C$C\underline{H_2}$ of Ad group), 1.78–1.56(7H, m, PC$\underline{H}$H, C$C\underline{H_2}$CH of Ad group), 1.35(3H, d, 7.2 Hz, CH(C$\underline{H_3}$)CONH$_2$), 1.10(3H, d, *J* 6.4 Hz, CHPCH$_2$CH(C$\underline{H_3}$)); ^{13}C NMR (100 MHz, $CDCl_3$): δ 175.8, 174.7, 156.6, 136.9, 136.8, 129.2, 129.1(2C), 128.5(2C), 128.1(2C), 127.9, 127.8, 126.7, 83.5, 67.1, 52.9, 49, 44.6(3C), 35.6(3C), 34.2, 31.2(3C), 30.7, 29.6, 17.7, 17.1; ^{31}P NMR(162 MHz, $CDCl_3$): 47.1, 46.1; LC-ESI–MS(+ve): *m/z* 610.7$[M+H]^+$.

[(*R/S*)-2-methyl-{3-(((*R*)-1-(amino)-2-phenylethyl)-adamantyloxyphosphinyl)propanoyl} amino]-(*S*)-2-propanamide (**5**).

To a solution of **4** (140 mg, 0.229 mmol) in MeOH (30 ml) 10% Pd/C (50 mg) was added and stirred in under 4 psi

© 2014 The Author(s)
The author(s) has paid for this article to be freely available under the terms of the Creative Commons Attribution Licence (CC-BY) (http://creativecommons.org/licenses/by/3.0/) which permits unrestricted use, distribution and reproduction in any medium, provided the original work is properly cited.

(1 psi = 6.9 kPa) of hydrogen for 4-6 h. The reaction mixture was filtered using celite, washed with EtOH (50 ml), and evaporation of solvent under vacuum affored [(*R/S*)-2-methyl-{3-(((*R*)-1-(amino)-2-phenylethyl)-adamantyloxyphosphinyl)propanoyl} amino]-(S)-2-propanamide **5** (82 mg, 75 %) as a yellow liquid.

^{1}H NMR(300 MHz, $CDCl_3$): δ 7.27-7.16(7H, m, Ar-H, CON$\underline{H_2}$), 5.44(2H, m, CHN$\underline{H_2}$), 4.49-4.30(1H, m, C$\underline{H}$CONH$_2$), 4.21-4.16(1H, m, C$\underline{H}$PCH$_2$), 3.22-3.11(1H, m, PCHCH$\underline{H}$Ph), 2.79-2.67(2H, m, PCHC$\underline{H}$HPh, PCH$_2$C$\underline{H}$), 2.31-2.22(1H, m, PCH$\underline{H}$CH), 2.14-2.07 (9H, m, C$\underline{H}$ of Ad group, CC$\underline{H_2}$ of Ad group), 1.78-1.56(7H, m, PC$\underline{H}$H, CC$\underline{H_2}$CH of Ad group), 1.35(3H, d, 7.2 Hz, CH(C$\underline{H_3}$)CONH$_2$), 1.10(3H, d, 6.4 Hz, CHPCH$_2$CH(C$\underline{H_3}$)); LC-ESI–MS(+ ve): *m/z* 476.3[M + H]$^+$.

[(*S*)-((3-1H-tetrazol-2-yl)-2-aminopropanoyl)-[(*S*)-2-methyl-{3-(((*R*)-1-(amino)-2-phenylethyl)-adamantyloxyphosphinyl) propanoyl}amino]]-(*S*)-2-propanamide (**33RE**).

To a solution of **5** (90 mg, 0.188 mmol, 1 equiv.) in dichloromethane (5 ml), EDC.HCl (39 mg, 0.207 mmol, 1.1 equiv.), 3-tetrazolyl-2-(1,1-dimethylethoxy)-methanamido propionic acid (53 mg, 0.207 mmol, 1.1 equiv.), HOBt (28 mg, 0.207 mmol, 1.1 equi.), DIPEA (0.066 ml, 0.376 mmol, 2 equiv.) were added at 0 °C. H_2O was added and the aqueous phase was extracted with ethyl acetate (30 ml×3). The combined organic layer was washed with H_2O (10 ml), brine (10 ml), dried over $MgSO_4$ and concentrated. The residue was treated with a mixture of TFA/DCM/anisole/$H_2$0 (4.5/0.4/0.1/0.1) for 3 h at room temperature. Evaporation of the solvent afforded the crude product which was chromatographed using reverse-phase HPLC to yield two diastereoisomers. The active component (**33RE**) of the above diastereisomeric mixture, corresponds to the first fraction that eluted and to 10 % of the total amount of tripeptide (t_R = 6.04).

^{1}H NMR(300 MHz, CD_3OD): δ 7.29-7.17(7H, m, Ar-H, CON$\underline{H_2}$), 4.86(2H, m, CHN$\underline{H_2}$), 4.51(1H, dd, 3.2 11.7 Hz, NH$_2$C$\underline{H}$ Tetrazole), 4.21(1H, t, *J* 4.7 Hz, C$\underline{H}$PCH$_2$), 4.09(1H, q, *J* 7.2 Hz, C$\underline{H}$CONH$_2$), 3.70(1H, dd, 5 15.9 Hz, NH$_2$CHCH$\underline{H}$ Tetrazole), 3.59(1H, dd, *J* 4.7 15.9 Hz, NH$_2$CHC$\underline{H}$H Tetrazole), 2.91-2.75(3H, m, PCHC$\underline{H_2}$Ph, PCH$_2$C$\underline{H}$), 2.28-2.17(1H, m, PCH$\underline{H}$CH), 1.77-1.66(1H, m, PC$\underline{H}$H), 1.31(3H, d, *J* 7.7 Hz, CH(C$\underline{H_3}$)CONH$_2$), 1.29(3H, d, *J* 7.3 Hz, CHPCH$_2$CH(C$\underline{H_3}$)); LC-ESI–MS(+ ve): *m/z* 481.6[M + H]$^+$.

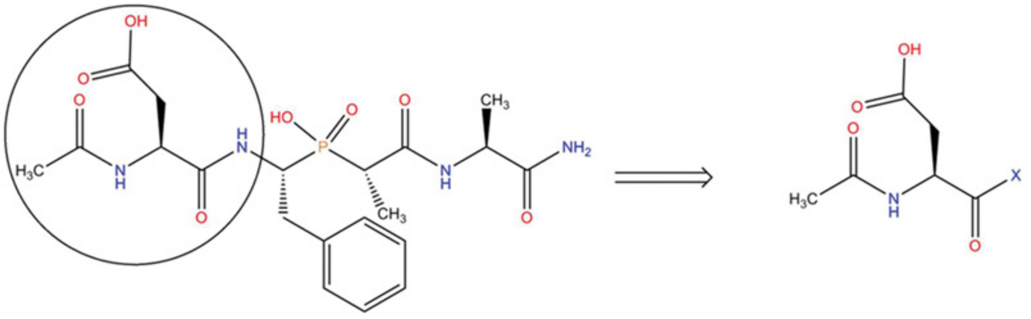

Figure S1 Query fragment that was used to define the region where the computation is performed

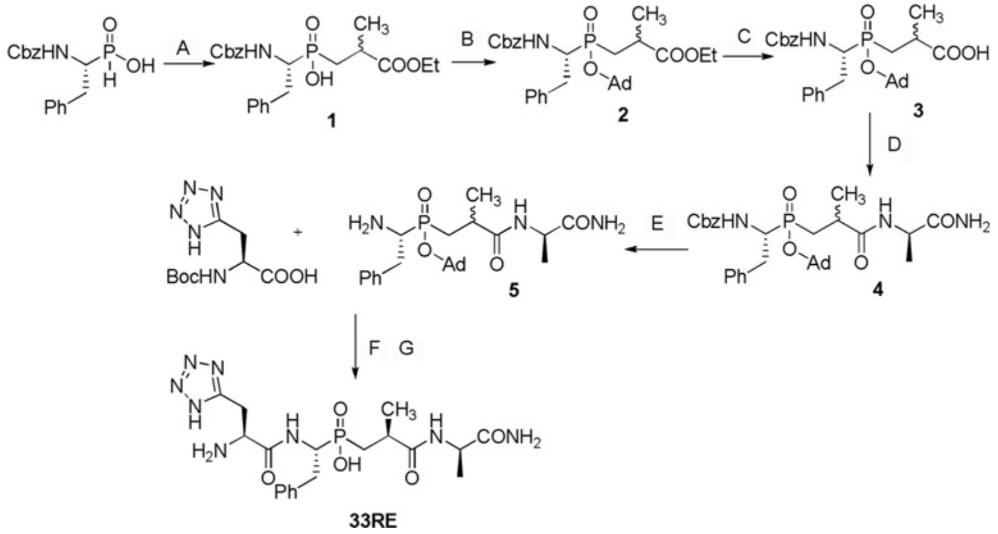

Scheme S1 Synthetic protocol for compound 33RE

Reactant and reagents: (A) ethyl methacrylate, HMDS, DIPEA, 110 °C, 5 h; (B) 1-adamantyl bromide, Ag_2O, $CHCl_3$, 5 h; (C) NaOH, MeOH, 2 h; (D) L-alaninamide, EDC.HCl, HOBt, DIPEA, DCM, 18 h; (E) 10 % Pd/C, H_2, 8 h; (F) EDC.HCl, HOBt, DIPEA, DCM, 18 h; (G) TFA/anisole/H_2O/DCM, 3 h.

© 2014 The Author(s)
The author(s) has paid for this article to be freely available under the terms of the Creative Commons Attribution Licence (CC-BY) (http://creativecommons.org/licenses/by/3.0/) which permits unrestricted use, distribution and reproduction in any medium, provided the original work is properly cited.

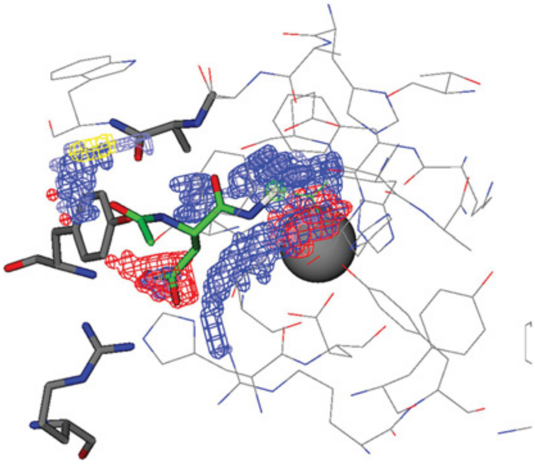

Figure S2 GRID molecular interaction fields that are computed in the region where the fragment is selected
The blue field is obtained by the N1 probe and represents H-bond donor regions, the red field is obtained using the O probe and represents H-Bond acceptor regions and the yellow field is obtained using the DRY probe and represents hydrophobic regions.

REFERENCES

1 Bergmann, R., Liljefors, T., Sorensen, M. D. and Zamora, I. (2009) SHOP: receptor-based scaffold HOPping by GRID-based similarity searches. J. Chem. Inf. Model **49**, 658–669

2 Anthony, C. S., Corradi, H. R., Schwager, S. L., Redelinghuys, P., Georgiadis, D., Dive, V., Acharya, K. R. and Sturrock, E. D. (2010) The N domain of human angiotensin-I-converting enzyme: the role of N-glycosylation and the crystal structure in complex with an N domain-specific phosphinic inhibitor, RXP407. J. Biol. Chem. **285**, 35685–35693

3 Kroger, W. L., Douglas, R. G., O'Neill, H. G., Dive, V. and Sturrock, E. D. (2009) Investigating the domain specificity of phosphinic inhibitors RXPA380 and RXP407 in angiotensin-converting enzyme. Biochemistry **48**, 8405–8412

4 Wei, L., Alhenc-Gelas, F., Corvol, P. and Clauser, E. (1991) The two homologous domains of human angiotensin I-converting enzyme are both catalytically active. J. Biol. Chem. **266**, 9002–9008

Received 19 July 2013/20 August 2013; accepted 9 September 2013

Published as Immediate Publication 9 September 2013, doi: 10.1042/CS20130403

© 2014 The Author(s)
The author(s) has paid for this article to be freely available under the terms of the Creative Commons Attribution Licence (CC-BY) (http://creativecommons.org/licenses/by/3.0/) which permits unrestricted use, distribution and reproduction in any medium, provided the original work is properly cited.
